# Supplementary material for: Sevoflurane versus ketamine+diazepam anesthesia for assessing systemic and hepatic hemodynamics in rats with non-cirrhotic portal hypertension
Source: PLoS One. 2020 May 29;15(5):e0233778. doi: 10.1371/journal.pone.0233778 (PMC7259621; doi:10.1371/journal.pone.0233778)
Supplement: Attachment — Submitted filename: Response to Reviewers.docx [file pone.0233778.s002.docx]

We would like to sincerely thank the editor and both reviewers for their comments and constructive criticisms. We hope to have adequately addressed them.

**Journal requirements**

1. *Please ensure that your manuscript meets PLOS ONE´s style requirements, including those for file naming.*

We have made some modifications in order to fully meet PLOS ONE´s style requirements. Specifically:

- Inclusion of a short title (Page 1)
- Figure captions have been inserted immediately after the first paragraph in which the figure was cited (Pages 14, 15, 16, 17 and 18)
- Tables have been inserted immediately after the first paragraph in which they were cited (Table 1: pages 6-7; Table 2: page 15).

2. *Ethics statement*
   a. *Please amend your current ethics statement to include the full name of the ethics committee that approved your specific study*
   b. *Please amend your current ethics statement to confirm that your name ethics committee specifically approved this study.*

We have amended our ethic statement by including the full name of the ethics committee that specifically approved our study: “Comité de Ética en Experimentación Animal of the Hospital General Universitario Gregorio Marañon and the Comunidad de Madrid (PROEX# 272/15)” (Page 9).

3. *Additional details in your Methods section regarding animal care*
   a. *Please state the source and number of mice used in the study.*

In this study, we used male Sprague-Dawley rats from Charles River Laboratories (France). We stated the source and number of animals (total and per group) in the first paragraph of page 9: *“Male Sprague-Dawley (SD) rats (310-580 g, n= 41) from Charles River Laboratories (France) were…”* and *“the following four groups of rats were evaluated: Ctrl+Sevo (n=8), Ctrl+KD (n= 11), PPVL+Sevo (n=9), and PPVL+KD (n=13)”.*

b. *Please describe the post-operative care received by the animals, including the frequency of monitoring and the criteria used to assess animal health and well-being.*

We have included the description of the post-operative care in the first paragraph of page 10: “…antiseptic ointment was applied to the surgical wound. Body temperature was monitored with a thermometer and maintained at normal levels with a warming pad throughout the operation. Once the animals recovered from the procedure, they were returned to individual cages, and physiological and behavioral signs indicative of pain or discomfort (body position, self-cleaning, body weight, motility, food intake) were assessed daily.”.

4. *Please include captions for your Supporting Information files at the end of your manuscript, and update any in-text citations to match accordingly.*

Captions for Supporting Information files have been included at the end of the manuscript (page 25), as indicated. A mention to the Supporting Information has also been included in the Statistical Analysis subsection of Material and Methods (page 12: “Raw data of the experiments are provided as Supporting Information (S1 File).”)

**Reviewer #1:**

Methods

1. *Number of animals per group is not homogeneous. Could authors comment on the possible mortality due to PPVL procedure or other reasons leading to such variance.*

No mortality due to the PPVL procedure was observed. The variability in the number of animals between groups was due to the increased mortality in the groups anesthetized with Ketamine-Diazepam (KD) for the hemodynamic study. In order to have similar group sizes with available hemodynamic data, we included more rats in both KD groups.

Results

2. *Portal pressure measurement. Authors observe no differences in PP values when comparing both groups of PPVL animals. This could be predicted considering that in the PPVL model a major contributor to the increment in the porto-vascular resistance (and therefore in PP) is the physical knot that indeed modifies the pre-hepatic resistance. Splanchnic hemodynamic measurement would be interesting to further assess the effects of both anesthetics. Could authors comment here and in the manuscript?*

We agree with Reviewer 1 that a major contributor to the increase in PP in the PPVL model is the increase in the pre-hepatic resistance caused by the physical knot. Although anesthesia cannot affect this “fixed resistance”, four days after the PPVL operation (time at which the hemodynamic study was performed) the hyperdynamic splanchnic and systemic circulation characteristic of portal hypertension are well established and contribute to the increase in PP (Sikuler et al, Am J Physiol. 1985;248: G618-625). The anesthetic regime may influence these other factors, and therefore affect PP values. Measurement of splanchnic flow as suggested by the reviewer would have provided valuable data concerning this issue.

We have added a comment in the 2^nd^ paragraph of Discussion (page 20): “Although no measurement of splanchnic flow was performed, the similar increase of portal pressure in PPVL rats suggests that both anesthetic regimes had a similar effect (or no effect) on the hyperdynamic splanchnic and systemic circulation induced by the PPVL, as pre-hepatic resistance was “fixed” by the PPVL operation.”.

3. *How authors interpret their findings on sevoflurane-associated mortality? Why was it higher in the ctrl vs the PPVL group?*

No mortality was observed in control and PPVL groups anesthetized with sevoflurane. As far as the higher KD-associated mortality found in the Ctrl vs the PPVL group is concerned, we cannot provide a solid explanation. We can speculate that it may be partially related to the unpredictable individual sensitivity to ketamine, which is unlikely to be influenced by the hemodynamic changes induced by the PPVL procedure. To confirm this hypothesis, however, would likely require very large sample-size of both groups.

Discussion

4. *Although this is an interesting and timely study, these same analyses would be desirable in pre-clinical models of cirrhotic portal hypertension where hepatic function is compromised and, probably, hepatic metabolism of anesthetics would play a bigger role. This limitation/future opportunity should be discussed in the manuscript. Also, I would suggest to include “non-cirrhotic portal hypertension” in the title and abstract of the manuscript.*

We agree with both reviewers in this issue. This limitation has been included in the 4th paragraph of Discussion (page 21):

“It is important to point out, however, that the PPVL model induces pre-hepatic portal hypertension, which limits the extrapolation of our findings to animal models of cirrhosis (e.g. carbon tetrachloride). Future studies would be needed to assess if the performance of the KD anesthetic regime is altered further by the potential pharmacokinetic and pharmacodynamic derangements induced by cirrhosis”.

We have also included “non-cirrhotic portal hypertension” in the title, abstract and throughout the manuscript.

**Reviewer #2:**

1. *The title and throughout the manuscript the authors should state that these are findings on non-cirrhotic portal hypertension, they cannot generalize those findings in cirrhotic models such as BDL and CCL4, this should be seen at first sight.*

We agree with both reviewers in this issue. This limitation has been included in the 4th paragraph of Discussion (page 21):

“It is important to point out, however, that the PPVL model induces pre-hepatic portal hypertension, which limits the extrapolation of our findings to animal models of cirrhosis (e.g. carbon tetrachloride). Future studies would be needed to assess if the performance of the KD anesthetic regime is altered further by the potential pharmacokinetic and pharmacodynamic derangements induced by cirrhosis”.

We have also included “non-cirrhotic portal hypertension” in the title, abstract and throughout the manuscript.

2. *The data presented as text, should be presented in tables, e.g. BW, duration of the measurements, biochemistry, and blood-gas-analysis.*

A new Table has been added with the data of BW, duration of the hemodynamic study and blood biochemistry. as suggested by the reviewer (Table 2, page 15). We preferred to leave arterial blood gas data in the text, as we presented compounded data of the Ctrl and PPVL groups of each anesthetic group.

3. *In Figure 1A and B the two different groups of each sevoflurane and Ketamine/Diazepam should be plotted besides each other not together.*

Figure 1 has been modified according to these recommendations.
